# Medicago *PHYA* promotes flowering, primary stem elongation and expression of flowering time genes in long days

**DOI:** 10.1186/s12870-020-02540-y

**Published:** 2020-07-11

**Authors:** Mauren Jaudal, Jiangqi Wen, Kirankumar S. Mysore, Joanna Putterill

**Affiliations:** 1 The Flowering Lab, School of Biological Sciences, University of Auckland, Auckland, New Zealand; 2 Noble Research Institute, 2510 Sam Noble Parkway, Ardmore, OK73401 USA

**Keywords:** *PHYA*, Photoperiodic flowering time, Medicago, Arabidopsis, Legume, *FTa1*, *E1*, *FUL*, *SOC1*, Primary stem elongation

## Abstract

**Background:**

Flowering time is an important trait for productivity in legumes, which include many food and fodder plants. *Medicago truncatula* (Medicago) is a model temperate legume used to study flowering time pathways. Like *Arabidopsis thaliana* (Arabidopsis), its flowering is promoted by extended periods of cold (vernalization, V), followed by warm long day (LD) photoperiods. However, Arabidopsis flowering-time genes such as the *FLOWERING LOCUS C* (*FLC*)/ *MADS AFFECTING FLOWERING* (*MAF*) clade are missing and *CONSTANS-LIKE* (*CO-LIKE*) genes do not appear to have a role in Medicago or *Pisum sativum* (pea). Another photoperiodic regulator, the red/far red photoreceptor PHYTOCHROME A (PHYA), promotes Arabidopsis flowering by stabilizing the CO protein in LD. Interestingly, despite the absence of CO-LIKE function in pea, PsPHYA plays a key role in promoting LD photoperiodic flowering and plant architecture. Medicago has one homolog of *PHYA*, *MtPHYA,* but its function is not known.

**Results:**

Genetic analysis of two *MtPHYA Tnt1* insertion mutant alleles indicates that *MtPHYA* has an important role in promoting Medicago flowering and primary stem elongation in VLD and LD and in perception of far-red wavelengths in seedlings. *MtPHYA* positively regulates the expression of *MtE1-like* (*MtE1L*), a homologue of an important legume-specific flowering time gene, *E1* in soybean and other Medicago LD-regulated flowering-time gene homologues, including the three *FLOWERING LOCUS T-LIKE* (*FT-LIKE)* genes, *MtFTa1, MtFTb1* and *MtFTb2* and the two *FRUITFULL-LIKE* (*FUL-LIKE*) genes *MtFULa* and *MtFULb. MtPHYA* also modulates the expression of the circadian clock genes, *GIGANTEA* (*GI)* and *TIMING OF CAB EXPRESSION 1a (TOC1a)*. Genetic analyses indicate that *Mtphya-1 Mte1l* double mutants flowered at the same time as the single mutants. However, *Mtphya-1 Mtfta1* double mutants had a weak additive effect in delaying flowering and in reduction of primary axis lengths beyond what was conferred by either of the single mutants.

**Conclusion:**

*MtPHYA* has an important role in LD photoperiodic control of flowering, plant architecture and seedling de-etiolation under far-red wavelengths in Medicago. It promotes the expression of LD-induced flowering time genes and modulates clock-related genes. In addition to *MtFTa1, MtPHYA* likely regulates other targets during LD floral induction in Medicago.

## Background

Legumes are the third largest group of plants and the second most important crops after the cereals, with flowering time a key factor in determining their productivity and yield [1–4]. *Medicago truncatula* (Medicago) is a model nitrogen-fixing legume and a diploid, self-fertile annual forage plant [5]. It is related to important temperate legume forages and foods including *Medicago sativa* (alfalfa), *Trifolium* species (clovers), *Pisum sativum* (garden pea), *Cicer arietinum* (chickpeas) and *Lens culinaris* (lentils). Major Medicago genomic resources include genome sequences and large collections of mutants including *Tnt1* retroelement insertion lines [5–7]. These are accompanied by a searchable flanking sequence tag database facilitating forward and reverse genetics to analyze flowering time control [6–9].

Like the winter annual ecotypes of *Arabidopsis thaliana* (Arabidopsis), Medicago is a temperate-climate plant whose flowering time is promoted by long day (LD) photoperiods and by vernalization – an extended exposure to winter cold. This ensures that flowering occurs in the lengthening warm days of spring only after winter has passed [10]. However, interestingly, the key regulatory genes known to be targets of the Arabidopsis vernalization pathway - the repressor *FLOWERING LOCUS C* (*FLC)* and associated *MADS AFFECTING FLOWERING (MAF)* clade are missing in Medicago [4, 8].

In addition, while CONSTANS (CO) has an important role in photoperiodic control of flowering in Arabidopsis activating the potent stimulator of flowering *FLOWERING LOCUS T* (*FT*) in leaves in LD, Medicago *CO-like* genes do not appear to be involved in flowering time control [11]. Mt *co-like Tnt1* insertion mutants flower like wild-type [11]. Similarly, the transcript levels of *MtCO-like* genes are not altered by the overexpression of a Medicago *CYCLING DOF FACTOR-like* gene, *MtCDFd1_1* that nevertheless leads to a delay in Medicago flowering in LD [12]. Similar results were obtained in the analysis of pea flowering time mutants that affect photoperiodic flowering and/or circadian clock function such as *late2* (*Pscdfc1)* [13], *late1* (*Psgigantea (gi*)) [14] and *dne* (*die neutralis*, *Pself4*) [4, 15]. These mutations alter pea *FT-like* gene expression, but not the expression of the closest pea relative to *CO*, *PsCOLa.*

Other candidate components of Medicago flowering-time pathways including *FT-LIKE* genes have also been investigated and are implicated in flowering time control. *MtFTa1* is a key regulator of flowering in Medicago. It is expressed predominantly in leaves where expression is elevated by exposure to the floral inductive conditions of vernalization followed by LD. In addition, *Mtfta1 Tnt1* insertion mutants were late flowering, while over expression of *MtFTa1* accelerated flowering [16]. The expression of *MtFTa1* and the two other LD-induced Medicago *FT-like* genes, *MtFTb1* and *MtFTb2,* was reduced in the late flowering *MtCDFd1_1* over expressing plants [12], while *MtFTa1* expression was precociously elevated in *spring* mutants that flower rapidly compared with wild type plants [17, 18].

The *MtE1L* gene is a Medicago homolog of the important *Glycine max* (soybean) photoperiodic flowering time regulator *E1,* a legume-specific gene [4, 19]*.* A *MtE1L* knock-out mutation caused a delay in flowering time in Medicago indicating a role in promoting flowering [19]. Functional analysis of the three *Mt SUPPRESSOR OF OVEREXPRESSION OF CONSTANS1* (*SOC1*)-*like* genes identified a late flowering *Mtsoc1a* mutant, supporting a role for *MtSOC1a* in promoting flowering [20]. Like *MtSOC1a*, *MtSOC1b* and *MtSOC1c* were photoperiodically regulated and were partly dependent on *MtFTa1* for their expression [20, 21]. All three *MtSOCs* can partially complement the late flowering of the *Atsoc1* mutant [21]. Two *FRUITFULL-LIKE (FUL-like)* genes, *MtFULa* and *MtFULb*, promoted flowering when over expressed in heterologous Arabidopsis and their expression was partly dependent on *MtFTa1,* indicating a likely role in flowering time control in Medicago [22]*.* On the other hand, *MtVERNALISATION2 (VRN2)* has a different function from that of Arabidopsis *VRN2*, because *MtVRN2* represses *MtFTa1* and flowering before vernalization [23]. In addition, while the two *MtSHORT VEGETATIVE PHASE-LIKE (SVP-like)* genes, *MtSVP1* and *MtSVP2*, repressed flowering when overexpressed in Arabidopsis, over expression of *MtSVP1* did not delay flowering in Medicago [24].

Medicago PHYTOCHROME A (PHYA) is a candidate photoperiodic flowering regulator whose function has not previously been reported. In Arabidopsis, this red/far red photoreceptor promotes flowering in LD by promoting CO protein accumulation in leaves. PHYA antagonizes the SUPPRESSOR of phyA-105/ CONSTITUTIVELY PHOTOMORPHOGENIC 1 (SPA/COP) complex, which degrades CO protein [25, 26]. PHYA also plays a role in the entrainment of the Arabidopsis circadian clock and thus, the circadian-regulated diurnal expression of LD flowering time genes such as *CO* and *GI* [27, 28]. Despite the apparent absence of a Medicago CO-like function [11], a role for *PHYA* in promoting temperate legume photoperiodic flowering is strongly supported by previous classical genetic studies in pea. Pea *phya* loss of function mutants cause strong LD-specific delays to flowering and display architectural phenotypes similar to plants grown in short day (SD) conditions [29]. The important role of *PsPHYA* in promoting flowering is also shown by a dominant *Psphya* mutation that confers *PHYA* stability and causes early flowering [30].

Here, to further investigate photoperiodic flowering control in Medicago, we carried out a reverse genetic analysis of the *PHYA* gene, which exists as a single copy in the Medicago genome [31, 32]. We report the effect of two independent Medicago *phya* mutations on flowering time and plant architecture. We examine the expression of candidate flowering time and clock genes in the *Mtphya-1* mutant in different environmental conditions and investigate genetic interactions by generating double mutant plants of *Mtphya* combined with either *Mtfta1* or *Mte1l*.

## Results

### Initial characterisation of *MtPHYA*

*MtPHYA* (Medtr1g085160) is predicted to encode a 1124 aa protein containing the important domains that comprise the N-terminal photosensory core module and the C-terminal regulatory region typical of PHYA-like proteins (Additional file 1: Figure S1) [27]. It is 79% identical to Arabidopsis PHYA, but shares highest homology with PHYA-like protein sequences from other temperate legumes including pea (95%), red clover (95%), chickpea (93%) and lotus (89%) (Additional file 1: Figure S1).

**Fig. 1:**
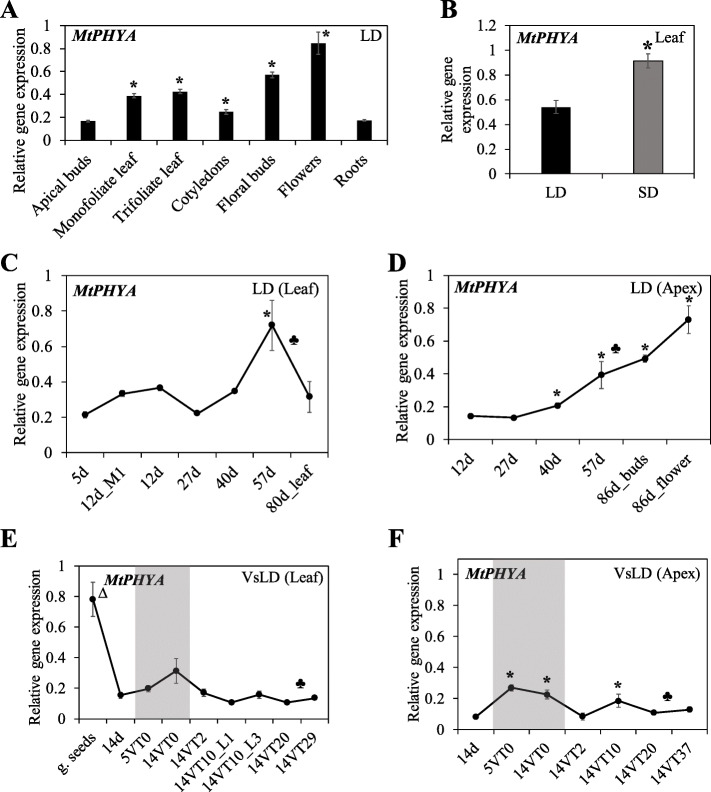
*MtPHYA* is expressed broadly and throughout development in Medicago wild type R108 plants. **a** Relative expression of *MtPHYA* in tissues of plants grown in long-day (LD) photoperiods. The cotyledons were from 5-d-old seedlings; monofoliate and trifoliate leaves, apical buds and roots from 12-d-old plants; and floral buds (young) and flowers from 86-d-old flowering plants. **b** Relative transcript abundance of *MtPHYA* in fully-expanded trifoliate leaves of 14-d-old seedlings grown under LD and short-day (SD) photoperiods. **c-d** Relative gene expression levels of *MtPHYA* in leaves (**c**) and uppermost apical buds, flower buds or open flowers (**d**) in days after planting in LD. The ♣ in (**c**-**d**) indicate that the plants flowered with floral buds first visible at 69 days. Cotyledons were harvested at 5 days while monofoliate leaves (M1) or trifoliate leaves were harvested at the remaining time points. **e-f***MtPHYA* expression in germinated seeds (g. seeds) and in leaves (**e**) and uppermost shoot apices (**f**) of plants before vernalization (14d), during vernalization at 4 °C (shaded) with sampling done after 5 days (5VT0 = 5 days in the cold, 0 day warm) and 14 days (14VT0), and after the plants were transferred to warm LD conditions (VsLD) (14VT2 = 2 days in the warm after 14 days of vernalization). The ♣ in (**e-f**) indicate that the plants flowered with floral buds first visible at 52 days after planting. The tissues in (**a-f**) were harvested two hours after dawn (ZT2). Gene expression was determined using qRT-PCR with primers 2F and 2R (Additional file 2: Table S1) and is shown as the mean ± se of three biological replicates, normalized to Medicago *PP2A* and relative to the highest value in (**a** and **b**). For **c**-**d** and **e**-**f** the data is shown relative to the highest value over both tissues. The * indicates significantly different expression from the first data point in (**a-d, f**) while the ∆ in (**e**) indicates that the expression in germinated seeds is significantly different from the rest of the data points [multiple pairwise comparisons adjusted for false discovery rate (FDR); α = 0.05]

**Fig. 2:**
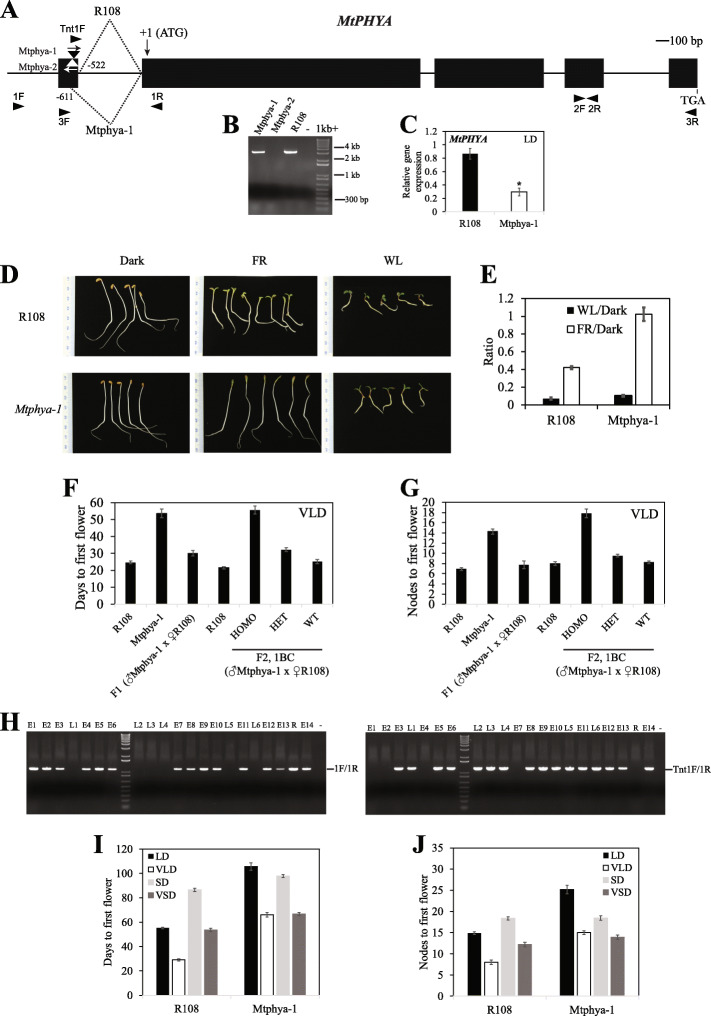
*The Mtphya-1* mutant has reduced sensitivity to far-red light and flowers later than wild type R108 particularly in LD and VLD photoperiods. **a***MtPHYA* with *Tnt1* insertions at − 536 bp and − 556 bp upstream of the ATG in the *Mtphya*-*1* (black triangle) and *Mtphya*-*2* (white triangle) mutants, respectively. Exons are black boxes and introns are thin lines. Arrows indicate orientation of *Tnt1* insertions. Dotted lines indicate splice sites used in *Mtphya*-*1* compared with R108. **b** cDNA fragments amplified by primers 3F and 3R. **c***MtPHYA* expression in 14-d-old seedlings in LD, 2 h after dawn, using qRT–PCR with primers 2F and 2R. Mean ± SE three biological replicates, normalized to Medicago *PP2A* and relative to the highest value. * significantly different expression from R108 using one-way analysis of variance (ANOVA) test between the means (α = 0.05). **d-e** Seedlings in white light (WL), far-red, FR and dark (**d**) and ratio of hypocotyl lengths of 3-d-old seedlings to dark-grown (**e**). Mean ± (t.SE) (0.05), *n* = 9. (**f-g**) Flowering time in vernalized LD (VLD) scored as days to flowering (**f**) or the number of nodes on the primary axis at flowering (**g**) of the F1 progeny (*n* = 8) from *Mtphya-1* crossed to R108, *Mtphya-1* (self-cross) (*n* = 21), R108 (*n* = 18) and segregating F2 progeny (*n* = 217: *Mtphya-1 Tnt1* homozygotes, *n* = 50; heterozygotes, *n* = 114; wild-type segregants, *n* = 53) with R108 (*n* = 25). Data are mean ± (t.SE) (0.05). (**h**) PCR genotyping fragments from segregating F2 plants in (F-G). Plants were scored as early (**e**) (like R108) or late (**l**) flowering relative to R108. 1F and 1R used for wild-type band and 1R and Tnt1F for *Tnt1*. **i-j** Graphs showing the flowering time in different conditions of *Mtphya-1* mutants (no backcross) and R108 scored in days (**i**) or nodes to first flower (**j**). Mean ± (t.SE) (0.05) is presented (*n* = 9–16)

We analysed *MtPHYA* gene expression in different tissues and environments by qRT-PCR using gene-specific primers 2F and 2R (Fig. 2a, Additional file 2: Table S1). *MtPHYA* was detected in a wide range of tissues 2 h after dawn, including cotyledons, leaves, apical buds, open flowers and roots (Fig. 1a) and was slightly more abundant in SD than in LD photoperiods (Fig. 1b). Analysis of *MtPHYA* expression in a developmental time-course in LD in leaves and shoot apices indicated that it did not change significantly in leaves, except for a 2-fold increase prior to flowering, but its expression declined after flowering (Fig. 1c). In contrast, in shoot apices, there was a steady increase in expression through development and it continued to rise after flowering (Fig. 1d).

Medicago flowering is promoted by prolonged winter cold (vernalization, V) followed by long-day (LD) photoperiods [10]. To assess if vernalization has a direct effect on *MtPHYA* transcript, we analysed its expression in a vernalized seedling long-day (VsLD) time course. Seeds were germinated, grown in LD, then 14-d-old seedlings were vernalized at 4 °C and afterwards returned to warm LD. *MtPHYA* was expressed in leaves and apices of seedlings before, during and after vernalization at similar levels, although at a significantly lower level than in germinated seeds (Fig. 1e-f). This indicates that *MtPHYA* expression is not directly regulated by cold. The high abundance of *MtPHYA* in etiolated germinated seeds (Fig. 1e), is consistent with other plants including pea where *PHYA* transcript accumulates to a much higher level in the dark than in the light [29, 30, 33].

### Two independent *Mtphya* mutant lines have reduced sensitivity to far-red light compared with wild type R108 seedlings

To investigate the function of *MtPHYA* in Medicago plant development, we analysed two independent *Tnt1* retrotransposon-tagged R108 mutant lines, *Mtphya-1* (NF1583) and *Mtphya-2* (NF3601), which have insertions facing in opposite directions in the 5′ UTR of *MtPHYA* (Fig. 2a). To analyse if the *Tnt1* insertions affected the full-length transcript of *MtPHYA*, we used primers 3F and 3R (Fig. 2a, Additional file 2: Table S1) to amplify cDNA fragments from wild type R108 and the two mutant lines. The primers amplified cDNA fragments from both R108 and *Mtphya-1*, but none from *Mtphya-2* indicating that the latter was a knockout mutant (Fig. 2b)*.* Direct sequencing of the PCR product from R108 indicated that the intron upstream of the ATG in R108 was spliced out using the 5′ splice donor site at position − 522 to generate a 3533 bp cDNA. However, sequencing of the PCR product from *Mtphya-1* indicated alternative splicing of this upstream intron, which utilized a 5′ splice donor site at position − 611. This led to splicing out of the *Tnt1* insertion and adjacent 89 bp in the 5’UTR of *Mtphya-1*, which resulted in amplification of a slightly shorter cDNA (3444 bp). As measured by qRT-PCR using primers 2F and 2R, *Mtphya-1* and *Mtphya-2* had a statistically significant ~ 3-fold and ~ 11-fold reduction, respectively, in *MtPHYA* gene expression, compared with wild-type R108 plants (Figs. 2c, 4a).

Because PHYA has a well-documented role in regulation of seedling photomorphogenesis [34, 35], we then analysed seedling de-etiolation responses of the two *Mtphya* mutant lines and wild-type R108 **(**Figs. 2d-e, 4b). Seeds were germinated in the dark overnight and then grown for three days either under far-red (FR) light, in the dark (D) or in white light (WL). Under continuous FR light, both the *Mtphya* mutants had longer hypocotyls with unexpanded cotyledons, compared to wild type R108 seedlings with short hypocotyls and expanded, green cotyledons **(**photographs shown for *Mtphya-1*, Fig. 2d). In contrast, in WL, the *Mtphya* mutants had short hypocotyls that were only slightly longer than wild-type R108 hypocotyls **(**photographs shown for *Mtphya-1*, Fig. 2d). In dark, the hypocotyls of both mutants were very long like dark-grown, wild-type R108 seedlings (photographs shown for *Mtphya-1,* Fig. 2d). When the hypocotyl lengths were plotted as a ratio of light to dark grown, the WL/Dark hypocotyl length ratios were low in the wild-type and both the *Mtphya* mutants (Figs. 2e, 4b). However, the FR/Dark hypocotyl length ratio in the *Mtphya* mutants was much higher than in R108 (Figs. 2e, 4b). These results indicate that the *Mtphya* mutants had reduced sensitivity to far-red light.

### *Mtphya* mutants flower later than wild type R108 particularly in LD and VLD photoperiods

To investigate if the *Mtphya* mutations affect flowering time*,* we characterized the mutant plants for their flowering time phenotypes compared with wild type R108 plants. First, under vernalized long day photoperiod (VLD) conditions, both *Mtphya-1* and *Mtphya-2* mutant plants were late flowering compared with R108 (Figs. 2f-g, 4c-d). We then crossed homozygous *Mtphya-1* mutants with wild-type R108 control plants and analysed the genotype and flowering time of the F1 plants and the segregating F2 population in VLD (Fig. 2f-h). The F1 progeny had a weak intermediate phenotype, flowering slightly later than wild-type R108, but much earlier than *Mtphya-1* mutants. In the segregating F2 population of 217 plants, ~ one quarter (*n* = 50) were *Mtphya-1 Tnt1* homozygotes and late flowering, ~ half (*n* = 114) were heterozygotes and flowered slightly later than wild-type R108 and wild-type segregants, and ~ a quarter (*n* = 53) were wild-type segregants and flowered like wild-type R108. Thus, the *Tnt1* insertion in *Mtphya-1* was tightly linked to the late flowering locus (within ≤1 cM).

The *Tnt1* insertion in *Mtphya-2* also showed 100% co-segregation with the late flowering phenotype. The pattern of inheritance in *Mtphya-2* was analysed by characterising the flowering time of a segregating population from heterozygous, self-crossed parents in VLD. Out of 45 plants, about one quarter (n = 11) were homozygotes and all were late flowering, and ~ one quarter (*n* = 8) were wild-type segregants and early flowering like wild-type R108. The remaining plants (*n* = 26) were heterozygotes, which showed semi-dominance as observed for *Mtphya-1,* because they displayed an intermediate late flowering time phenotype (Fig. 4c-d).

To further investigate the role of *MtPHYA* in regulation of flowering, *Mtphya-1* plants were grown under different photoperiodic conditions, with or without, vernalization treatment of germinated seeds (Fig. 2i-j). As expected, the R108 wild-type plants exhibited a strong response to photoperiod and vernalization, flowering most rapidly in vernalized long day (VLD) but flowered the latest under non-vernalized short day (SD) conditions. However, the *Mtphya-1* mutants were strikingly impaired in their ability to respond to LD compared with wild type plants. *Mtphya-1* mutants were delayed in flowering in both LD and VLD compared to R108, but flowered at a similar time to wild type R108 in VSD and SD. The mutants exhibited a late flowering day-neutral phenotype, particularly in vernalized conditions, as they flowered at a similar time in VLD and VSD. The *Mtphya-1* mutants retained the ability to respond to vernalization because they flowered earlier in VLD than LD, and similarly, the VSD-grown mutants were earlier than the SD-grown plants. However, the response of *Mtphya-1* to vernalization was slightly weaker compared with R108.

### *Mtphya* mutants have a very short primary axis in LD and VLD photoperiods compared with wild type

**Fig. 3:**
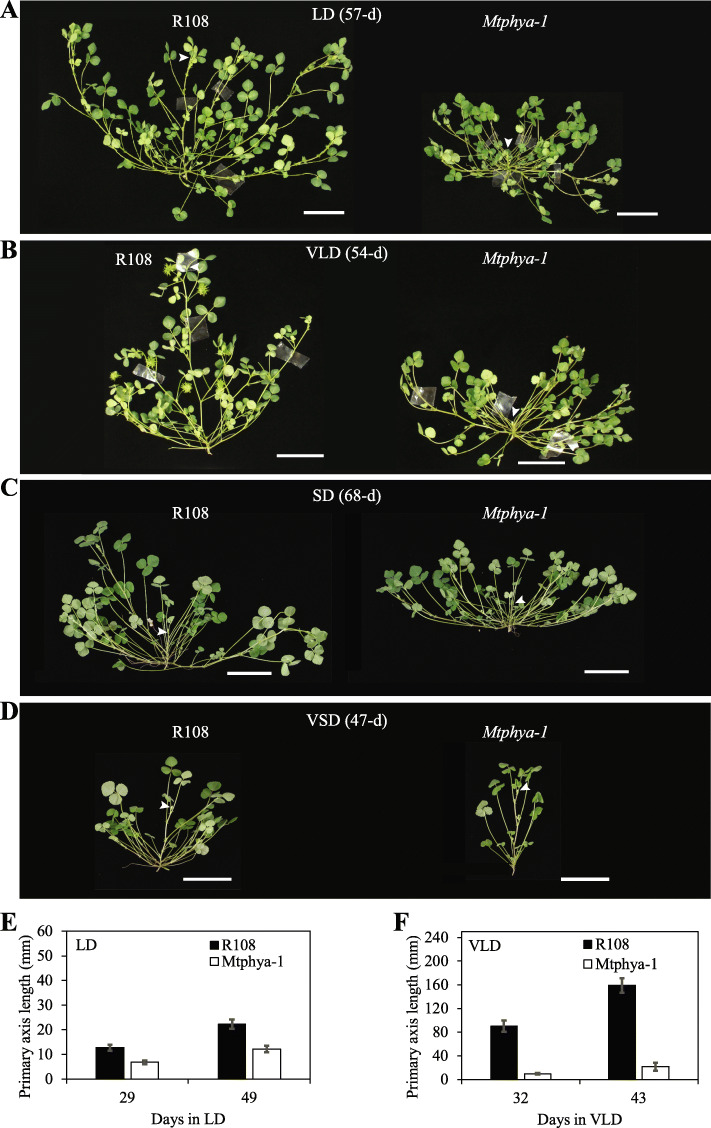
*The Mtphya-1* mutant has a very short primary axis in LD and VLD compared to R108. **a-d** Photographs of wild type R108 and *Mtphya-1* (non-backcross) mutants in LD (**a**), VLD (**b**), SD (**c**) and VSD (**d**). Arrowheads indicate the primary axis. Scale bar = 5 cm. **e-f** Graphs showing the lengths of the primary axis of wild-type R108 and *Mtphya-1* mutants at different days of growth in LD (**e**) and VLD (**f**). The LD *Mtphya-1* plants were F3 homozygous mutants after a backcross to R108 while the VLD *Mtphya-1* plants were F2 homozygous mutants after two backcrosses to R108. The data are shown as the mean ± (t.SE) (0.05) (n = 5–10)

**Fig. 4:**
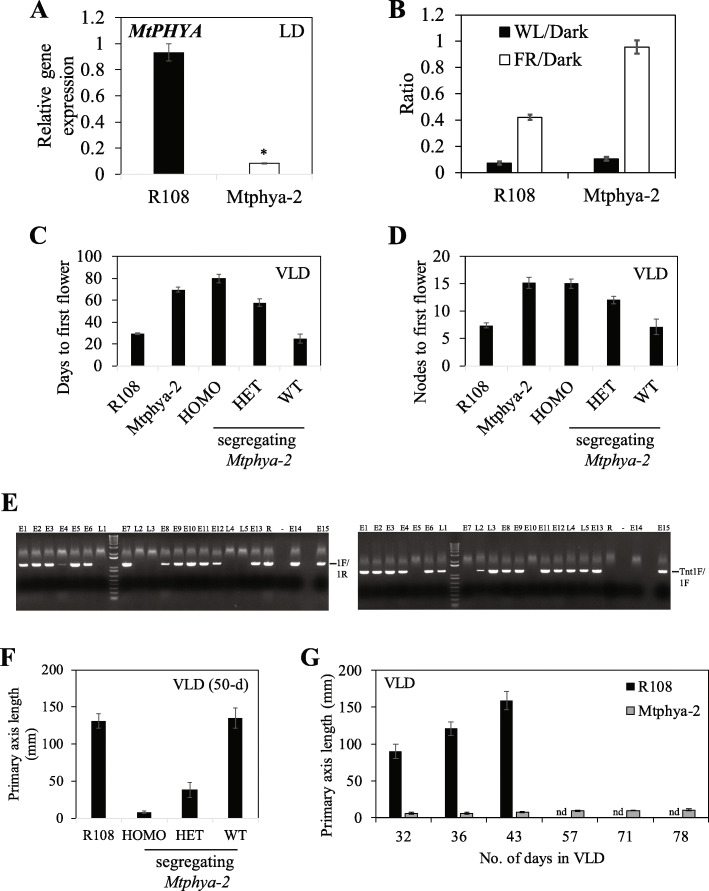
The *Mtphya-2* mutant has reduced sensitivity to far-red light, is late flowering and has a very short primary axis throughout development in VLD compared to R108. **a** Relative expression of *MtPHYA* in 25-d-old plants in LD 2 h after dawn using qRT–PCR with 2F and 2R primers. Data are the mean ± SE of three biological replicates, normalized to Medicago *PP2A* and relative to the highest value. The * indicates significantly different expression from wild type R108 using one-way ANOVA test between the means (α = 0.05). **b** Ratio of hypocotyl lengths of 3-d-old seedlings of R108 and *Mtphya-2* in different light conditions (white light, WL; far-red, FR) to dark-grown. Data are mean ± (t.SE) (0.05), *n* = 9. **c-d** Graphs showing VLD flowering time in days (**c**) or the number of nodes on the primary axis at flowering (**d**) of a segregating population of *Mtphya-2* heterozygous plants (self-crossed) (*n* = 45: *Mtphya-2* homozygotes, n = 11; heterozygotes, *n* = 26; wild-type n = 8) compared with *Mtphya-2* mutants (self-crossed) (*n* = 6) and R108 plants (*n* = 10). Data are the mean ± (t.SE) (0.05). **e** PCR genotyping fragments from segregating *Mtphya-2* plants in (**c-d**). Plants scored as early (E) (like R108) or late (L) flowering relative to R108. Genotyping primers 1F and 1R (Fig. 2A) were used for wild-type band and primers 1F and Tnt1F for *Tnt1* insertion. **f** Primary axis lengths in VLD of segregating *Mtphya-2* plants in (**c-e**) compared with R108. Measurements are the mean ± (t.SE) (0.05) (n = 8–26). **g** Lengths of primary axis of R108 and *Mtphya-2* over time in VLD. *Mtphya-2* mutants were homozygous F2 plants after backcrossing to R108. Data are mean ± (t.SE) (0.05) (n = 8–10). R108 plants flowered at 23–26 d while *Mtphya-2* mutants at ~ 65–71 d in VLD. nd, not done

In addition to displaying delayed flowering, both the *Mtphya* mutant plants were more compact than wild type R108, with a strikingly short primary axis (see photographs of *Mtphya-1* mutant in Fig. 3a-b). This phenotype was more pronounced in LD photoperiods (both LD and VLD) than in SD or VSD conditions in the *Mtphya-1* mutant compared with R108 (Fig. 3a-d). Measurements of primary axis lengths, taken at different stages of development, indicated that both the *Mtphya* mutants exhibited the short axis phenotype compared to R108, prior to and after flowering (Fig. 3e-f, Fig. 4g). To analyse the inheritance of the short primary axis phenotype, we also measured primary axis length in VLD in the population segregating for the *Mtphya-2* mutation (Fig. 4f), previously analysed for flowering time (Fig. 4c-d). The short axis phenotype was only observed in the *Mtphya-2* homozygous segregants (Fig. 4f). Thus, there was co-segregation between the short primary axis phenotype and the late flowering phenotype (Fig. 4c-d). These results indicate that the late flowering time defect and the short primary axis phenotype are both caused by mutations in the *MtPHYA* gene.

### The delayed flowering of *Mtphya-1* in LD and VLD is associated with a decrease in expression of LD-induced *MtFTs*, *MtFULs* and *MtE1L*

**Fig. 5:**
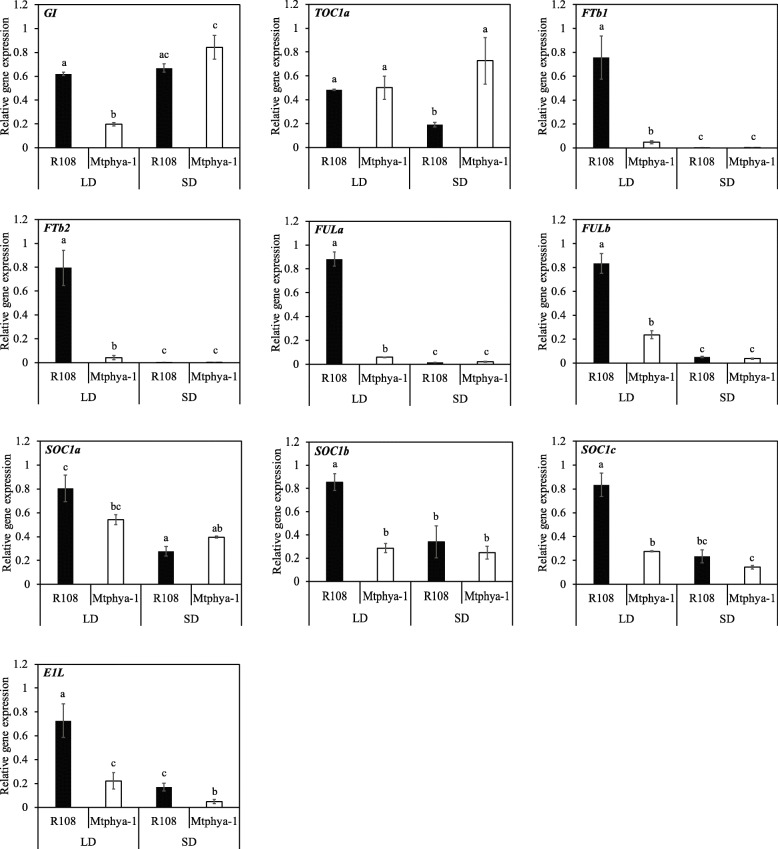
Gene expression of candidate photoperiod and flowering time genes in the *Mtphya-1* mutant and R108 in LD and SD. Relative transcript abundance was measured in the fully expanded trifoliate leaves of 14-d-old wild-type R108 and *Mtphya-1* homozygous seedlings. Relative gene expression was measured by qRT-PCR with normalization to *PP2A*. Data are the mean ± se of three biological replicates and relative to the highest value. Tissues were harvested 2 h after dawn. Different letters (**a,b,c**) indicate significant differences, while the same letter(s) on a bar indicate no significant differences using the two-way ANOVA test [multiple pairwise comparisons adjusted for false discovery rate (FDR); α = 0.05]. Tissues were harvested 2 h after dawn

**Fig. 6:**
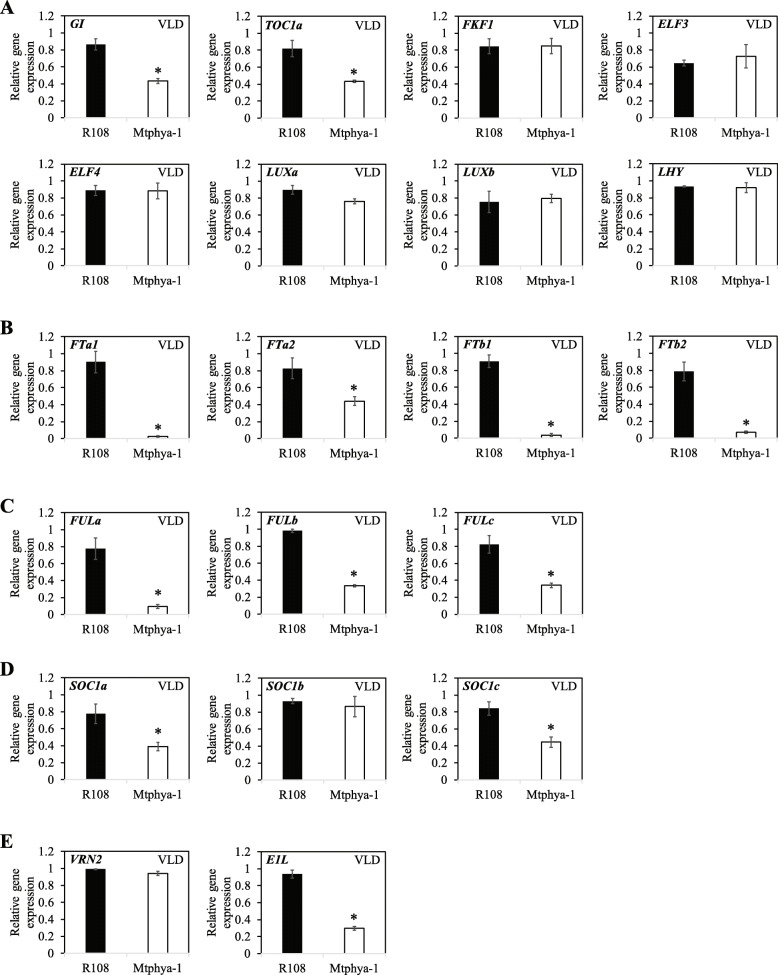
Gene expression of candidate circadian and flowering time genes in the *Mtphya-1* mutant and R108 in VLD. Relative transcript abundance was measured in the fully expanded trifoliate leaves of 14-d-old wild-type R108 and *Mtphya-1* homozygous seedlings grown in vernalised long-day (VLD) photoperiods. Relative gene expression was measured by qRT-PCR with normalization to *PP2A*. Data are the mean ± t.se of three biological replicates and relative to the highest value. The * indicates significantly different expression from wild type R108 using one-way ANOVA test between the means (α = 0.05). Tissues were harvested 2 h after dawn

Next, we analysed the molecular basis of the altered long day photoperiod flowering time response of *Mtphya-1* mutants. To do this, we analysed the expression of candidate Medicago circadian clock and flowering-time genes in leaves of the *Mtphya-1* mutant and wild type plants in LD and SD (Fig. 5) and in VLD (Fig. 6) two hours after dawn (ZT2) using qRT-PCR.

The expression level of *GI* was similar in LD and SD in wild type R108 and was reduced by ~ 3-fold in the *Mtphya-1* mutants compared with R108 in LD, but not in SD (Fig. 5). *GI* was reduced by ~ 2-fold in the mutant in VLD (Fig. 6). *TOC1a* level increased by ~ 4-fold in the *Mtphya-1* mutant in SD compared with R108, but was unchanged in LD (Fig. 5) at ZT2. However, it was reduced by ~ 2-fold in the mutant in VLD compared with R108 control (Fig. 6a). The other Medicago clock-related genes homologous to *ELF3, ELF4, LUXa, LUXb* and *LHY* were analysed only in VLD, but were not changed compared with wild type R108 (Fig. 6a**)**. The expression of a candidate flowering-time gene, *FKF1*-*like*, was also not changed in the mutant compared with wild type in VLD (Fig. 6a).

The expression of the three LD-induced *MtFT* genes, *MtFTa1*, *MtFTb1* and *MtFTb2* [16] were strikingly reduced in the *Mtphya-1* mutant. *MtFTb1* and *MtFTb2* were highly expressed in LD but undetected under SD in R108, consistent with previous findings [16]. However, both genes were strongly decreased by ~ 16- and ~ 19-fold, respectively, in the *Mtphya-1* mutant compared with wild type in LD (Fig. 5). In VLD, *MtFTa1*, *MtFTb1* and *MtFTb2* were strongly decreased by ~ 39-, 24- and 11-fold, respectively in the *Mtphya-1* mutants compared with wild-type R108 (Fig. 6b). In contrast, the fourth *FT-like* gene tested, *MtFTa2*, was weakly reduced by ~ 2-fold in the mutant in VLD (Fig. 6b).

*MtFULb* was previously shown to be under photoperiodic control because it was expressed at higher levels in LD than in SD [17]. A similar result was observed here (Fig. 5). *MtFULa* was also photoperiodically regulated with strongly elevated expression in the leaves of wild type R108 under LD compared with SD (Fig. 5). The *Mtphya-1* mutation had a particularly strong effect on the expression of *MtFULa* because it was decreased by ~ 15-fold, while *MtFULb* was reduced by ~ 3.5-fold in the *Mtphya-1* mutant compared with wild type in LD. Similarly, in VLD, *MtFULa* was reduced by ~ 8.3-fold and *MtFULb* by ~ 3-fold in the *Mtphya-1* mutant compared with wild type R108 (Fig. 6c).

The three *MtSOC1* genes were expressed at higher levels in LD than in SD in R108 as shown in previous work [20, 21]. However, the expression of *MtSOC1a* was not significantly changed in the leaves of the *Mtphya-1* mutants compared with R108 in either LD or SD conditions. *MtSOC1b* and *MtSOC1c* decreased by ~ 3-fold in the mutant in LD but were unchanged in SD (Fig. 5). In VLD, the *Mtphya-1* mutants showed a weak decrease in the expression of *MtSOC1a* and *MtSOC1c* (~ 2-fold) but not in *MtSOC1b* compared with R108 (Fig. 6d).

We observed that *MtE1L* is ~ 3.5 fold more abundant in LD than in SD in R108, indicating that it is also photoperiodically controlled (Fig. 5). The same pattern was observed for the expression of *MtE1L* in the *Mtphya-1* mutant. However, the mutant plants showed a reduction in *MtE1L* transcript level by ~ 3-fold in LD and SD compared with R108 control and a similar pattern was observed under VLD (Fig. 6e).

There was no significant change in the transcript level of *MtVRN2* [23] in the leaves of *Mtphya-1* mutants compared with wild type (Fig. 6e).

### *Mtphya-1 Mte1l* double mutants flower at the same time as the *Mtphya-1* mutant in VLD

**Fig. 7:**
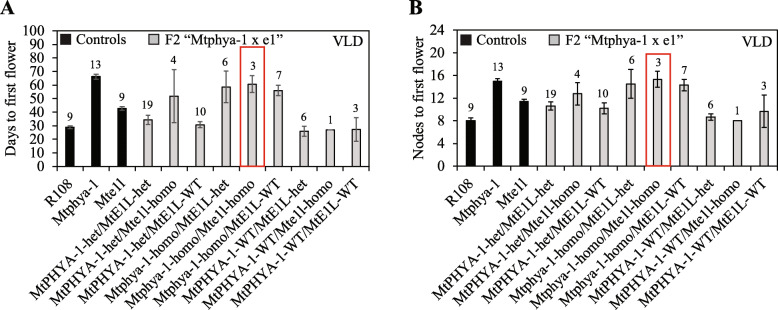
*Mtphya-1 e1l* double mutants flower at the same time as the *Mtphya-1* single mutant in VLD. Average flowering time of controls and the F2 population of *Mtphya-1* x *Mte1l* in VLD. Flowering time was scored as either days (**a**) or nodes (**b**) to first flower and are shown as the mean ± (t.SE) (0.05). The numbers of plants scored of each genotype are shown above the bars. The box indicates the double *Mtphya-1 Mte1l* mutant plants

Since *MtE1L* expression is under LD photoperiodic control and its level decreased in the *Mtphya-1* mutant, we tested genetically if *MtE1L* and *MtPHYA* promoted flowering via a common pathway. The *Mte1l* mutant line (NF16583) with a *Tnt1* insertion in the *MtE1L* coding region was obtained [19]*.* The *Mte1l* single mutants were moderately delayed in flowering compared with wild-type R108 controls in VLD (Fig. 7). We then crossed *Mtphya-1* with *Mte1l* and genotyped and scored the flowering time of the segregating F2 population from the cross. Three out of 59 (~ 1/16) F2 plants were *Mtphya-1 Mte1l* double mutants. These plants flowered late in VLD at a similar time to the single *Mtphya-1* mutants (Fig. 7). Therefore, no additive effect on flowering time was seen indicating that *Mte1l* and *Mtphya-1* were likely to be in the same pathway. The other *Mtphya-1* homozygous plants, either wild type or heterozygous at *MtE1L,* were also similarly delayed in flowering comparable to *Mtphya-1* mutant (Fig. 7). However, only a very weak effect on flowering time was seen in the one *MtPHYA* wild type/*Mte1l* homozygous F2 plant obtained, consistent with the mild effect of *Mte1l* on flowering in VLD (Fig. 7).

### *Mtphya-1 fta1* double mutants flower slightly later than either of the single mutants in VLD

Plants with a knockout mutation at *MtFTa1* are late flowering with short primary axes and prostrate architecture [16], similar to the *Mtphya* mutants. In addition, *MtFTa1* expression is strongly reduced in the *Mtphya-1* mutant in VLD. Thus, to test if *MtFTa1* and *MtPHYA* promote flowering in the same flowering time pathway, we crossed *Mtphya-1* and *fta1* mutants to generate a double mutant. In our initial experiment, we obtained six F2 plants, but genotyping indicated that none were double mutants. Therefore, a F2 *Mtphya-1*-homozygous/ *FTa1*-heterozygous plant was selected, self-crossed, and its F3 progeny were genotyped and scored.

**Fig. 8:**
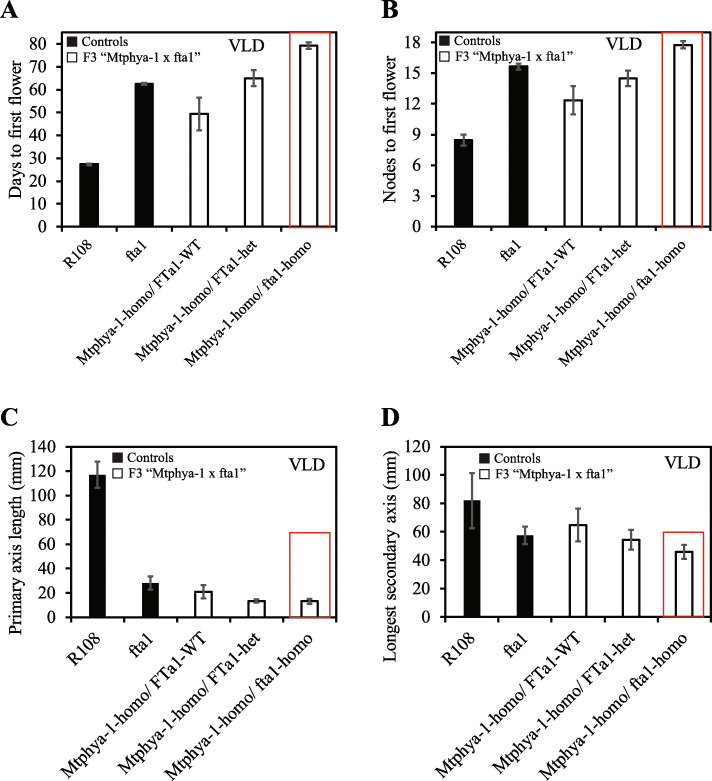
*Mtphya-1 fta1* double mutants show weakly additive effects on flowering time and primary axis length in VLD. **a-b** Double *Mtphya-1 fta1* mutants were generated by crossing the single mutants. Average flowering time in VLD of controls (wild-type R108, n = 9; *fta1* mutants, *n* = 14) and F3 progeny of a *Mtphya-1* homozygous/*FTa1* heterozygous plant from *Mtphya-1* x *fta1* (*n* = 48: *Mtphya-1* homozygous/*FTa1* wild-type, *n* = 15; *Mtphya-1* homozygous/*FTa1* heterozygous, *n* = 24; *Mtphya-1* homozygous/*fta1* homozygous, n = 9). Flowering time was scored as either days (A) or nodes (B) to first flower and are shown as the mean ± (t.SE) (0.05). The box indicates the double *Mtphya-1 fta1* mutant plants. **c-d** Average lengths of the primary (**c**) and longest secondary axis (**d**) of the plants in (**a**-**b**). The measurements were taken at 38-d-old in VLD and shown as the mean ± (t.SE) (0.05) (n = 9–24)

We identified 9 *Mtphya-1 fta1* double mutants out of 48 F3 plants. They flowered later (by ~ 2 weeks and with 3–4 more nodes) than the *Mtphya-1*-homozygous F3 plants (Fig. 8a-b). They also flowered later than *Mtfta1* single mutants grown as controls (Fig. 8a-b)*.* This indicates that the mutations at both loci caused a weak additive effect compared with the single mutants in VLD. In addition, the 24 *Mtphya-1* homozygous/*FTa1* heterozygous plants flowered slightly later than the *Mtphya-1* homozygous/*FTa1* wild-type plants (Fig. 8a-b). This suggests that *fta1* and *Mtphya-1* largely affect flowering in the same pathway, but also possibly under different pathways. Apart from the delay in flowering time, mutations at both loci also caused a weak additive effect on reduction of the primary axis length (Fig. 8c), with a slight reduction in the length of the longest secondary axis (Fig. 8d).

## Discussion

PHYA photoreceptors regulate photoperiodic flowering in several plants including Arabidopsis, the temperate legume pea and the tropical legumes soybean and common bean [28, 29, 36–38]. Here, we demonstrate using reverse genetics that *MtPHYA* has a major role in LD photoperiodic regulation in Medicago. It is required for the promotion of Medicago flowering by LD and VLD, but has little effect under SD and VSD. The *Mtphya-1* mutant had a late-flowering day-neutral phenotype compared with wild type R108 plants, particularly under vernalized conditions because the mutants flowered at the same time under VLD and VSD.

*Mtphya* mutants also displayed a very short primary axis early on development in LD and VLD and this was maintained even after flowering, in contrast to wild type plants. Thus, our work implicates *MtPHYA* in promoting primary axis elongation in Medicago in LD and VLD. Although the role of PHYA in seedling de-etiolation under continuous FR light is well characterized in many plant systems [29, 34, 35, 39, 40], there are exceptions. For example, in some legume species where the *PHYA* gene has been duplicated, genes including the *PHYA-like, PHOTOPERIOD* (*PPD*) in common bean [38] and the *E3* gene in soybean [36] do not perform this role. In Medicago, our results indicate that *MtPHYA* regulates Medicago seedling de-etiolation under FR light.

The role of PHYA photoreceptors in regulating flowering time through their effect on *FT* expression has been shown in tropical legume plants such as soybean [41] and common bean [38] but not reported previously for temperate legumes. Analysis of Medicago *fta1* mutants [16] and *Mtphya* mutants indicated similarities in their phenotype, including late flowering and prostrate plant architectures with very short primary axes. Moreover, the delay in flowering time of the *Mtphya* mutants in VLD correlated with a very strong reduction in *FTa1* transcript levels compared with wild type plants. The flowering time of *Mtphya-1 fta1* double mutants in VLD showed a weak additive effect in delaying flowering compared with the late flowering single mutants. Thus, although *MtPHYA* strongly positively regulates *FTa1* expression, *MtPHYA* might also regulate other flowering time targets.

Among the possible gene targets are *MtE1L*, *MtFTb1, MtFTb2, MtFULa* and *MtFULb* which were all induced by LD photoperiods [16, 18, 22] (this work) and were also decreased in the leaves of *Mtphya-1* mutants in VLD and LD compared with wild type plants. *MtFTb1* and *MtFTb2* function is not yet known, but their expression does not depend on *MtFTa1* [16]. Interestingly, in addition, *MtFULa* and *MtFULb* transcript levels appeared to be more strongly reduced in the *Mtphya-1* mutant than in the *Mtfta1* mutant plants relative to wild type [22]. These results support the idea that there may also be *FTa1*-independent pathways influenced by *MtPHYA*, *MtFULs* and *MtFTbs* in LD and VLD-mediated promotion of flowering and/or primary stem elongation in Medicago.

*E1* is a major regulator of photoperiodic flowering in soybean and is regulated by soybean *PHYA*, raising the possibility that *MtPHYA* might regulate Medicago flowering time via the *MtE1L* homologue. Previous analysis of the *Mte1l* mutant and wild type plants indicated that *MtE1L* promotes flowering in VLD [19]. Here, we showed that *MtE1L* transcript was photoperiodically regulated being more abundant in LD than in SD. *MtE1L* was reduced in *Mtphya-1* mutants compared with wild type R108 under LD, SD and VLD conditions suggesting that *MtE1L* is regulated by *MtPHYA*. The double *Mte1l Mtphya-1* mutants flowered at a similar time as the single *Mtphya-1* mutants in VLD, suggesting that *Mte1l* and *Mtphya-1* promote flowering in the same pathway in VLD. However, in contrast to soybean *E1*, *MtE1L* does not appear to have a major non-redundant role in promoting flowering in VLD in Medicago, because we only observed a moderate delay in flowering in the *Mte1l* mutants relative to R108 wild type plants.

The effect of *Mtphya* mutations on expression of candidate circadian clock has not been previously reported for pea or other temperate legumes. Here, most candidate circadian-clock related genes we analysed in Medicago did not vary in expression between the *Mtphya-1* mutant and wild type at the time point analysed in VLD. However, *MtGI* transcript levels were modestly but consistently reduced in *Mtphya-1* in both VLD and LD compared with R108. These results suggest that *MtPHYA* may regulate *MtGI* expression during the relay of the LD photoperiod signal that accelerates flowering in Medicago.

## Conclusions

Medicago is a long day plant, but the molecular basis of this photoperiodic regulation is still unclear because Medicago lacks a CO function, which has a central role in LD-mediated acceleration of flowering in Arabidopsis. Here, we demonstrate using reverse genetics that *MtPHYA* has an important role in LD photoperiodic control of flowering and aspects of plant architecture including primary stem elongation and seedling de-etiolation under far-red wavelengths in Medicago. *MtPHYA* promotes the expression of LD-induced flowering time genes and modulates expression of clock-related genes. In addition to *MtFTa1, MtPHYA* likely regulates other candidate targets in the LD and VLD floral induction pathway in Medicago. Thus, additional genomic and reverse genetic analyses could be carried out in the future to uncover the role of *MtGI*, *MtFTb1*, *MtFTb2*, *MtFULa* and *MtFULb* in *MtPHYA* LD-induced flowering.

## Methods

### Plant materials and growth conditions

*Medicago truncatula* (Medicago) wild-type R108_C3 (R108) [42] was used in this study. The R108 seeds were originally obtained from Dr. Pascal Ratet (Centre National de la Recherche Scientifique, Institut des Sciences du Vegetal, Gif-sur-Yvette, France). The seeds of the two *Tnt1* insertion mutant alleles for *MtPHYA*, *Mtphya-1* (NF1583) and *Mtphya-2* (NF3601) (this work), the *Tnt1* mutant for *FTa1* (*fta1*, NF3307) [16] and the *Tnt1* mutant for *MtE1L* (*Mte1l,* NF16583) [19] in the R108 background were obtained from the Noble Research Institute (Ardmore, OK, USA) [6].

Medicago plants were grown in controlled environments under ~ 200 μM m^− 2^ s^− 1^ cool white fluorescent light at 22 °C in long days (LD) (16 h light/8 h dark) or short days (SD) (8 h light/16 h dark) with or without prior vernalization of germinated seeds at 4 °C for 21 days, as previously described [12]. Flowering time was measured in either the total number of days after planting, or the total number of nodes along the primary axis at the time the first floral bud was observed by eye.

Homozygous *Mtphya-1* plants were crossed with homozygous *Mte1l* plants and then bred and genotyped to identify *Mtphya-1 Mte1l* double F2 mutants. Similarly, homozygous *Mtphya-1* was crossed with plants homozygous for *fta1* to generate *Mtphya-1 fta1* double mutants. Since, no double mutants were obtained from the segregating F2 plants, they were isolated from the F3 progeny of a *Mtphya-1*-homozygous/ *fta1*-heterozygous F2 plant. Genotyping was done using gene-specific and *Tnt1* primers (see Additional file 2: Table S1).

### RNA extraction, cDNA synthesis and quantitative reverse transcriptase PCR (qRT-PCR)

RNA extraction, cDNA synthesis using an oligo dT primer and qRT-PCR were carried out as previously described [12, 16, 18, 22]. Each data point represents the mean of three biological replicates harvested in parallel, with each replicate consisting of a pool of tissues from at least three independent plants at the 3-leaf stage of development (~ 14-d-old), unless indicated otherwise. Leaf and shoot apices (hand-dissected by eye) were harvested separately. The identity of the PCR amplicons was checked by DNA sequencing. Gene expression was normalized to *PROTEIN PHOSPHATASE 2A* (*PP2A*) (Medtr6g084690). The relative gene expression was calculated based on the 2^-ΔΔ**CT**^ method [43] with modifications [44] and normalised to the highest value as previously described [20]. Primers are shown in Additional file 2: Table S1.

The statistical testing for the gene expression data was performed using the one-way and the two-way analysis of variance (ANOVA) tests between the means (α = 0.05). The Shapiro-Wilk normality assumption test was performed on all the data presented. Multiple pairwise comparisons adjusted for False Discovery Rate (FDR) was utilised to highlight statistically significant differences in the data presented.

### Hypocotyl elongation tests

Seeds were scarified, sterilized and germinated in water overnight at 15 °C with shaking in the dark. Germinated seeds were transferred into ½ MS agar tubs with 8 g/L Kalys agar, pH 5.8 and placed in the dark (D), continuous white light (WL), ~ 100 μM m^− 2^ s^− 1^ or in far-red (FR) light at 22 °C for three days. For the FR light setting, a spectral wavelength of ~ 730 nm and intensity of ~ 1.7 μM m^− 2^ s^− 1^ was used.

## Supplementary information

**Additional file 1 Figure S1** Sequence alignment of PHYA-like proteins from temperate legumes and Arabidopsis.pdf. The deduced amino acid sequences were aligned using the MUSCLE plugin available in the Geneious software package [version 11.1.5 (http://www.geneious.com/)]. The domains highlighted include the N-terminal extension (**NTE**), Per-Arnt-Sim (**PAS**), cGMP phosphodiesterase/ adenylate cyclase/FhlA (**GAF**), and phytochrome (**PHY**), which comprise the N-terminal photosensory core module. The C-terminal regulatory module consists of the PAS-related domain (PRD) containing two PAS repeats (**PAS-A** and **PAS-B**) and the histidine kinase-related domain (**HKRD**) [domains adopted from 27]. At: *Arabidopsis thaliana*, Ca: *Cicer arietinum* (chickpea), Lj: *Lotus japonicas* (Lotus), Mt: *Medicago truncatula,* Ps: *Pisum sativum* (pea), Tp: *Trifolium pratense* (red clover). Identical and similar residues are highlighted in black.

**Additional file 2.** Table S1. List of primers.pdf.

## Data Availability

All data generated or analysed during this study are included in this published article and its supplementary information files. The Medicago *Tnt1* insertion lines are available from the Noble Research Institute, LLC.

## Abbreviations

LD: Long day
SD: Short day
VLD: Vernalized long day
VSD: Vernalized short day
qRT-PCR: quantitative reverse transcriptase PCR
FR: Far-red
WL: White light
D: Dark
FDR: False discovery rate
ANOVA: Analysis of variance
