# Supplementary material for: Medicago PHYA promotes flowering, primary stem elongation and expression of flowering time genes in long days
Source: BMC Plant Biol. 2020 Jul 11;20:329. doi: 10.1186/s12870-020-02540-y (PMC7353751; doi:10.1186/s12870-020-02540-y)
Supplement: Supplementary file 1 — Additional file 1 Figure S1 Sequence alignment of PHYA-like proteins from temperate legumes and Arabidopsis.pdf. The deduced amino acid sequences were aligned using the MUSCLE plugin available in the Geneious software package [version 11.1.5 (http://www.geneious.com/)]. The domains highlighted include the N-terminal extension (NTE), Per-Arnt-Sim (PAS), cGMP phosphodiesterase/ adenylate cyclase/FhlA (GAF), and phytochrome (PHY), which comprise the N-terminal photosensory core module. The C-terminal regulatory module consists of the PAS-related domain (PRD) containing two PAS repeats (PAS-A and PAS-B) and the histidine kinase-related domain (HKRD) [domains adopted from 27]. At: Arabidopsis thaliana, Ca: Cicer arietinum (chickpea), Lj: Lotus japonicas (Lotus), Mt: Medicago truncatula, Ps: Pisum sativum (pea), Tp: Trifolium pratense (red clover). Identical and similar residues are highlighted in black. [file 12870_2020_2540_MOESM1_ESM.pdf]

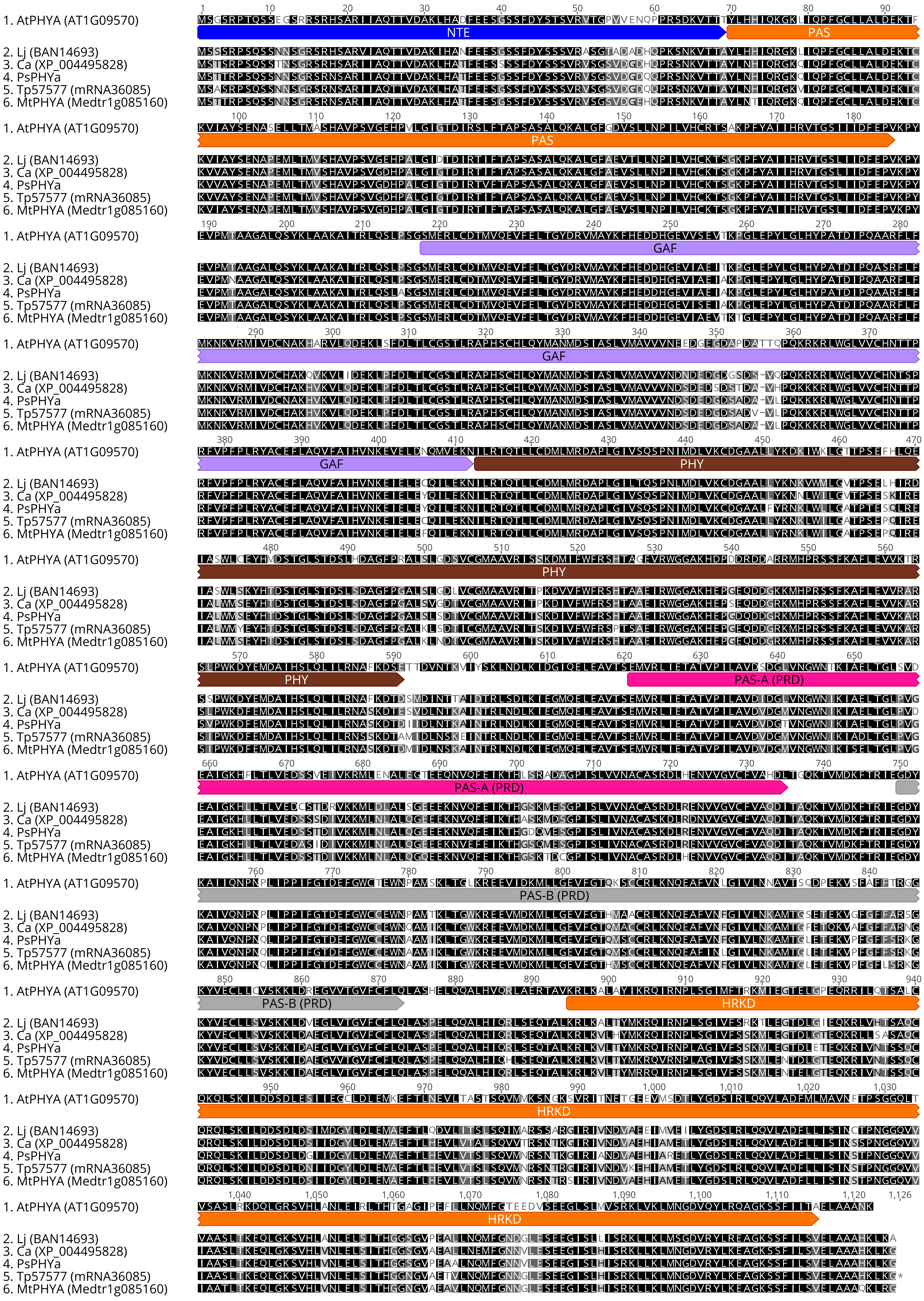
1. AtPHYA (AT1G09570)
2. Lj (BAN14693)
3. Ca (XP_004495828)
4. PsPHYa
5. Tp57577 (mRNA36085)
6. MtPHYA (Medtr1g085160)
NTE
PAS
GAF
PHY
PAS-A (PRD)
PAS-B (PRD)
HRKD

Additional file 1: Figure S1. Sequence alignment of PHYA-like proteins from temperate legumes and Arabidopsis. The deduced amino acid sequences were aligned using the MUSCLE plugin available in the Geneious software package [version 11.1.5 (http://www.geneious.com/)]. The domains highlighted include the N-terminal extension (**NTE**), Per-Arnt-Sim (**PAS**), cGMP phosphodiesterase/ adenylate cyclase/FhlA (**GAF**), and phytochrome (**PHY**), which comprise the N-terminal photosensory core module. The C-terminal regulatory module consists of the PAS-related domain (PRD) containing two PAS repeats (**PAS-A** and **PAS-B**) and the histidine kinase-related domain (**HKRD**) (domains adopted from Li et al. 2011). At: *Arabidopsis thaliana*, Ca: *Cicer arietinum* (chickpea), Lj: *Lotus japonicas* (Lotus), Mt: *Medicago truncatula*, Ps: *Pisum sativum* (pea), Tp: *Trifolium pratense* (red clover). Identical and similar residues are highlighted in black.
