# Supplementary material for: Medicago PHYA promotes flowering, primary stem elongation and expression of flowering time genes in long days
Source: BMC Plant Biol. 2020 Jul 11;20:329. doi: 10.1186/s12870-020-02540-y (PMC7353751; doi:10.1186/s12870-020-02540-y)
Supplement: Supplementary file 2 — Additional file 2. Table S1. List of primers.pdf. [file 12870_2020_2540_MOESM2_ESM.pdf]

Additional file 2:Table S1. List of primers.

Gene	Purpose	Primer ID	Sequence
MtPHYa	*Tnt1* line genotyping	1F	GAAAAAGGAAGGAAAGTCAAGGA
		1R	CTCCCTGAGTTGTTGGATGA
	expression	2F	GCATATCGCGATGGAAACCTTG
		2R	ACCTGACCTCCATTTGGTGTG
	checking cDNA	3F	GCTGGGTGGGAGACAAACAA
		3R	TTTTGGGCTGCCGCAAGTTC
E1	expression and *Tnt1* genotyping	F	ACAAGGGATCAGTGTCCACG
		R	CACCAAATCTGCAGCCAACA
ELF3	expression	F	AAAGGTTCAACTTGCCACCC
		R	TCAGGAGTTAAATGACCAGGGA
ELF4	expression	F	ACATGGTGAAGAACGTGGGT
		R	GAACGTTGTTGCTGGTGACA
FKF-1	expression	F	TCAACTGGGTATCGTGCTCA
		R	AACGACTGGATCCACCAAAG
FTa1	expression and *Tnt1* genotyping	F	GTAGCAGTAGGAATCCACTAGC
		R	ACACTCACTCTCGGTTGATTTCC
FTa2	expression	F	AAGTGGTAGCAGACCGAATC
		R	CACCACCATTGGTAAC
FTc	expression	F	GTTATGGTGGACGCAGATGC
		R	CAAATCGATGAATCCCTGCT
FTb1	expression	F	ATGAACCCTCTTGTGGTCTG
		R	TGGATTGACTATTTGGGAAG
FTb2	expression	F	ACAAATCCTCTTGTTGTTGG
		R	TGAGTTGATTATTTGAGAGG
FULa	expression	F	GGCCCAACTTGAGCAGCAAAATGAGG
		R	TGGGCGTTGCCATGGGTTTGAC
FULb	expression	F	AGAGCACGCAAAACTCAAGGCT
		R	AGCTCTTTGAGACCTAAACCATCCAA
FULc	expression	F	AGGGCAAGGACATTGCAGGAGCA
		R	TGGTGGTAGCACCTCTGGCTGACAA
GI	expression	F	AAACCTTTTGAAGTGTCGTCTAGCA
		R	GAGACGCTCAGAGCACGGACATG
LHY	expression	F	GAGGAGCATAAAGATGAGGAAAG
		R	CCGAAGATACAGATGAACAAGG
LUXa	expression	F	GTGCCTCTCGAAACACACTT
		R	TCGATCGGGTTCTTCGTCTT
LUXb	expression	F	AACCACCACCTTCTTCTCTTCC
		R	TTTGAGTGTTCGAGCTGGCT
SOC1a	expression	F	GCGTTGTTCGAGCAAGAAAGAATCAGGC
		R	GGGGCTGCTTAGAGAGCCTGGCATTT
SOC1b	expression	F	TCCAGAAACAAGATCAAGGCGCA
		R	TGATGAGACATTTGCCTCACCCTT
SOC1c	expression	F	TGCCATGCTCGCAGAGAAGT
		R	TCAGTTTCCACATCTGAACTTGGACT
Tnt1	*Tnt1* line genotyping	F	ACAGTGCTACCTCCTCTGGATG
		R	CAGTGAACGAGCAGAACCTGTG
TOC1a	expression	F	AGCAAGAGTGGTGATGGATTCA
		R	TGCCGTGCGGATTTTACAGA
PP2A	expression	F	GTGTTTTGCTTCCGCCGTT
		R	CCAAATCTTGCTCCCTCATCTG
VRN2	expression	F	TGTTCCGTGGGCTTGTGAGGCAT
		R	TGCCACTGTCTGATCCCCCGTTT
